# Electrostatically Enhanced Buried Interface Binding of Self‐Assembled Monolayers for Efficient And Stable Inverted Perovskite Solar Cells

**DOI:** 10.1002/adma.202508740

**Published:** 2025-08-11

**Authors:** Chuying Huang, Yi Yang, Cheng Liu, Hao Chen, Subhajyoti Chaudhuri, Woo Cheol Jeon, Muzhi Li, Nicholas Rolston, Abdulaziz S. R. Bati, Isaiah W. Gilley, Boran Kumral, Peter Serles, Tobin Filleter, George C. Schatz, Mercouri G. Kanatzidis, Bin Chen, Lin X. Chen, Edward H. Sargent

**Affiliations:** ^1^ Department of Chemistry Northwestern University 2145 Sheridan Rd Evanston IL 60208 USA; ^2^ Department of Electrical and Computer Engineering University of Toronto 35 St George Street Toronto ON M5S 1A4 Canada; ^3^ Department of Electrical and Computer Engineering Northwestern University 2145 Sheridan Rd Evanston IL 60208 USA; ^4^ Ira A. Fulton Schools of Engineering Arizona State University Tempe AZ 85281 USA; ^5^ Department of Mechanical and Industrial Engineering University of Toronto Toronto ON M5S 3G8 Canada

**Keywords:** interfacial adhesion, perovskite solar cells, self‐assembled monolayer, stability

## Abstract

Inverted p‐i‐n structure perovskite solar cells (PSCs) have outperformed traditional n‐i‐p PSCs in recent years. A key advancement is the use of self‐assembled monolayers (SAMs) as hole transport layers. One class of widely used SAMs is carbazole‐based phosphonic acids. However, it is found that these SAMs lack strong binding with transparent conducting oxides (TCO) and perovskite. The weak binding strength results in suboptimal interfacial adhesion of the buried interface, which limits the device's stability. Here, interfacial binding is enhanced by increasing the dipole moment that creates a strong interfacial electric field that enhances electrostatic interactions at the TCO/perovskite interface, while incorporating tailored functional groups in SAMs to improve chemical anchoring to TCO and binding to perovskite. Specifically, the donor‐acceptor SAM molecule 4‐(7‐(4‐(bis(4‐methoxyphenyl)amino)‐2,5‐difluorophenyl)benzo[c][1,2,5]thiadiazol‐4‐yl)benzoic acid (PAFTB) is employed, which features an enhanced dipole moment along with electron‐donating and electron‐withdrawing functional groups to optimize interfacial interactions. Compared to extensively used [2‐(9H‐carbazol‐9‐yl)ethyl]phosphonic acid (2PACz), PAFTB enhances total interfacial adhesion by 2.8 times, thereby improving the thermal stability of the layer. Using this approach, PSCs are demonstrated with a certified quasi‐steady‐state power conversion efficiency of 24.9% and maintain 80% of the initial efficiency after 900 h of maximum power point tracking at 85 °C.

## Graphical abstract

In p‐i‐n structure perovskite solar cells, conventional self‐assembled monolayers (SAMs) such as [2‐(9H‐carbazol‐9‐yl)ethyl]phosphonic acid (2PACz) exhibit limited buried interfacial adhesion, compromising device stability. A donor‐acceptor SAM, 4‐(7‐(4‐(bis(4‐methoxyphenyl)amino‐2,5‐difluorophenyl)benzo[c][1,2,5]thiadiazol‐4‐yl)benzoic acid (PAFTB), featuring an enhanced dipole moment and tailored functional groups, improves adhesion by 2.8 times, thereby enhancing both stability and efficiency of the devices.

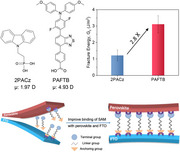

## 1 Introduction

Inverted perovskite solar cells (PSCs) have attracted attention for their efficiency, durability, low‐temperature processing, and compatibility with tandem structures.^[^ ^1^, ^2^, ^3^, ^4^, ^5^, ^6^ ^]^ Self‐assembled monolayers (SAMs) have contributed to this, aided by their tunable energy levels, low parasitic absorption, and defect passivation abilities, enabling their effective use as hole transport layers (HTLs) in inverted PSCs.^[^ ^7^, ^8^, ^9^, ^10^ ^]^ The molecules used in SAMs consist of anchoring, linker, and terminal groups. Among these, carbazole‐based phosphonic acids, such as [2‐(9H‐carbazol‐9‐yl)ethyl]phosphonic acid (2PACz) and [4‐(3,6‐dimethyl‐9H‐carbazol‐9‐yl)butyl]phosphonic acid (Me‐4PACz), have been widely employed in state‐of‐the‐art devices.^[^ ^8^, ^11^, ^12^ ^]^ However, the ultrathin SAMs at the buried interface raise concerns regarding device stability.^[^ ^13^, ^14^, ^15^, ^16^, ^17^, ^18^ ^]^

Recent studies have revealed several mechanisms underlying SAM‐related device degradation: i) insufficient binding between SAMs and substrates leads to weak interfacial adhesion and interface delamination;^[^ ^13^, ^14^, ^16^ ^]^ ii) poor interaction between SAM molecules causes instability of the HTL, degrading the quality of the interface;^[^ ^19^, ^20^ ^]^ iii) the lack of passivating functional groups in SAM molecules leads to the acceleration of defect‐induced perovskite degradation at the buried interface under aging conditions.^[^ ^21^, ^22^, ^23^, ^24^ ^]^ There remains room to explore and improve the reliability of the buried interface of SAM‐based devices.

We hypothesize that strengthening the binding of SAMs with both transparent conductive oxide (TCO) substrates and perovskite layers can suppress molecular desorption and enhance the stability of the buried interface. We sought to establish such a robust buried interface by using a SAM molecule that functions as a “molecular glue”.^[^ ^14^, ^16^ ^]^ Our strategy leverages dipole tuning and binding affinity optimization, improving the molecule's interaction with both TCOs and perovskites. SAM molecules with larger dipole moments are particularly advantageous, as their greater charge separation enhances electrostatic interactions and increases binding affinity to adjacent layers.^[^ ^25^, ^26^ ^]^ The anchoring strength can be further improved by incorporating special functional groups that strengthen chemical binding. In addition, integrating a conjugated donor‐*𝜋*‐acceptor (D‐𝜋‐A) structure into SAM molecules can ensure proper dipole orientation for hole transport, enhance carrier extraction, and reduce charge recombination.^[^ ^27^, ^28^, ^29^, ^30^ ^]^

Based on the above criteria, we introduced a D‐𝜋‐A type molecule with a high dipole moment and multiple functional groups, 4‐(7‐(4‐(bis(4‐methoxyphenyl)amino)‐2,5‐difluorophenyl) benzo[c][1,2,5]thiadiazol‐4‐yl) benzoic acid (PAFTB), in this study.^[^ ^28^, ^31^, ^32^ ^]^ We demonstrated that the high dipole D‐𝜋‐A structure and multiple functional groups in PAFTB play a crucial role in improving both the TCO/HTL and perovskite/HTL interfacial interactions, thereby improving the overall stability of PSCs. We demonstrated that PAFTB as the HTL enabled a power conversion efficiency (PCE) of 25.6% (certified quasi‐steady state PCE of 24.9%) with T_80_ of maximum power point tracking under 1‐sun at 85 °C for 900 h.

## 2 Results

### 2.1 Molecules and Interfacial Adhesion

**Figure 1:**
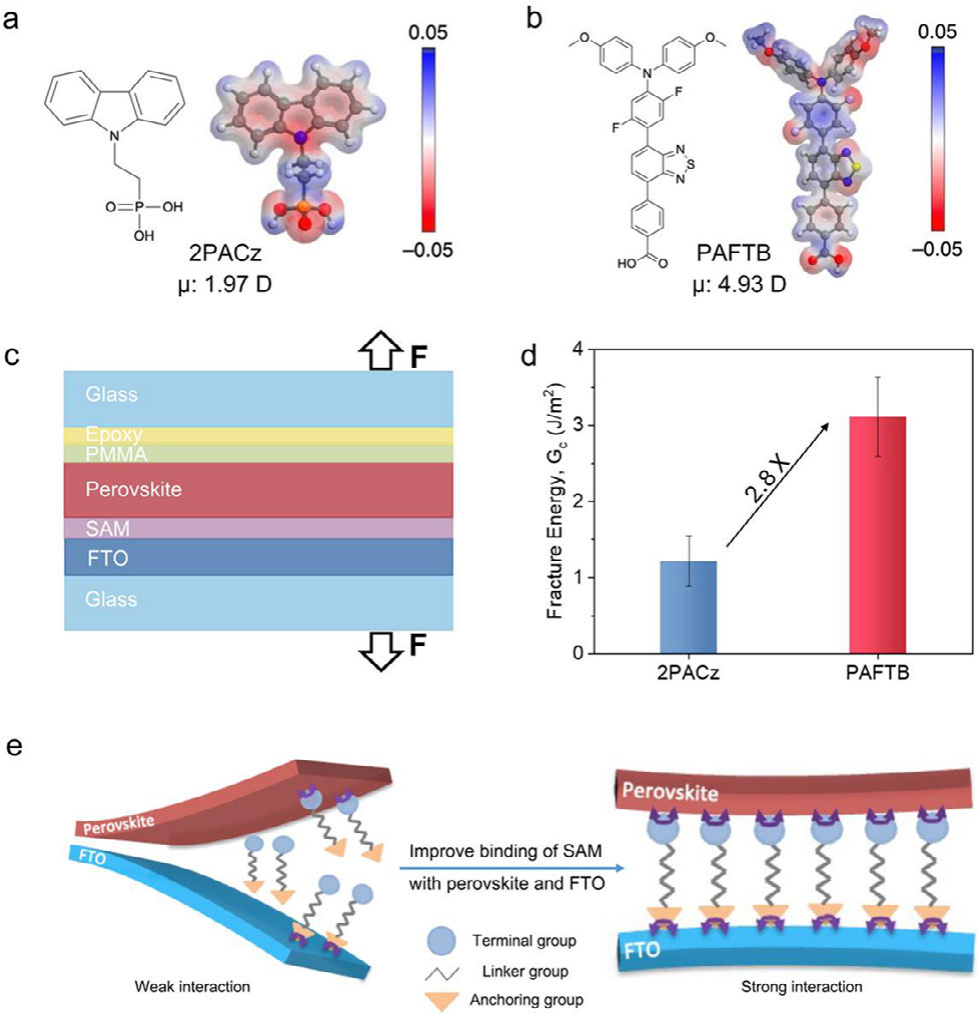
Investigation of molecular binding and interfacial adhesion. a) Electrostatic potential and molecular structure with dipole moment of 2PACz. b) Electrostatic potential and molecular structure with dipole moment of PAFTB. c) Schematic illustration of the interfacial adhesion test. The stack is glass/FTO/SAM/perovskite/PMMA/Ag/epoxy/glass. d) Fracture energy or interfacial adhesion (G_c_) of FTO/ perovskite with 2PACz and PAFTB at the interface. e) Schematic of a robust buried interface with SAM binding strongly with FTO and perovskite.

We began by evaluating the dipole moment and electrostatic potential (ESP) of SAM molecules by performing density functional theory (DFT) calculations (**Figure** **1**a,b). PAFTB exhibits a dipole moment 2.5 times larger than 2PACz, indicating its greater potential to provide stronger interactions with both fluorine‐doped tin oxide (FTO) and perovskite. These electrostatic interactions can be further strengthened by induced dipole moments once the molecules are adsorbed on the surface.^[^ ^33^, ^34^ ^]^ The ESP calculations also show that, compared to 2PACz, multiple functional groups, such as the carboxyl, methoxy, fluoro, and thiadiazole groups on PAFTB enable it to have various electron‐rich and electron‐deficient centers that can potentially passivate undercoordinated sites on the perovskite surface.

We examined the robustness of the buried interface by measuring the interfacial adhesion (G_c_) using the sandwich double‐cantilever beam (DCB) delamination method (Figure 1c).^[^ ^35^, ^36^ ^]^ The mechanical failure occurs at the weakest interface, and in this case, this is found to be the buried FTO/perovskite interface.^[^ ^37^ ^]^ The corresponding load‐displacement curves are shown in Figure S1 (Supporting Information). The samples based on 2PACz, which has been widely used in the state‐of‐the‐art inverted PSCs, showed an average G_c_ of 1.2 ± 0.3 J m^−2^. In contrast, PAFTB‐based samples demonstrated a 2.8‐fold increase in the average G_c_ (3.1 ± 0.5 J m^−2^) (Figure 1d). Cross‐section SEM images show similar buried interfaces for both PAFTB‐ and 2PACz‐based samples, which reflects comparable bond density of SAMs (Figure 4a; Figure S2, Supporting Information). The robustness of the buried interface G_c_ is dependent on bond density and bond strength. The higher G_c_ observed in PAFTB‐based samples arises from stronger bond strength, which is attributed to electrostatic interactions driven by the increased dipole moment of PAFTB compared to 2PACz (Figure 1e).

### 2.2 Strong Binding Between SAM and FTO

**Figure 2:**
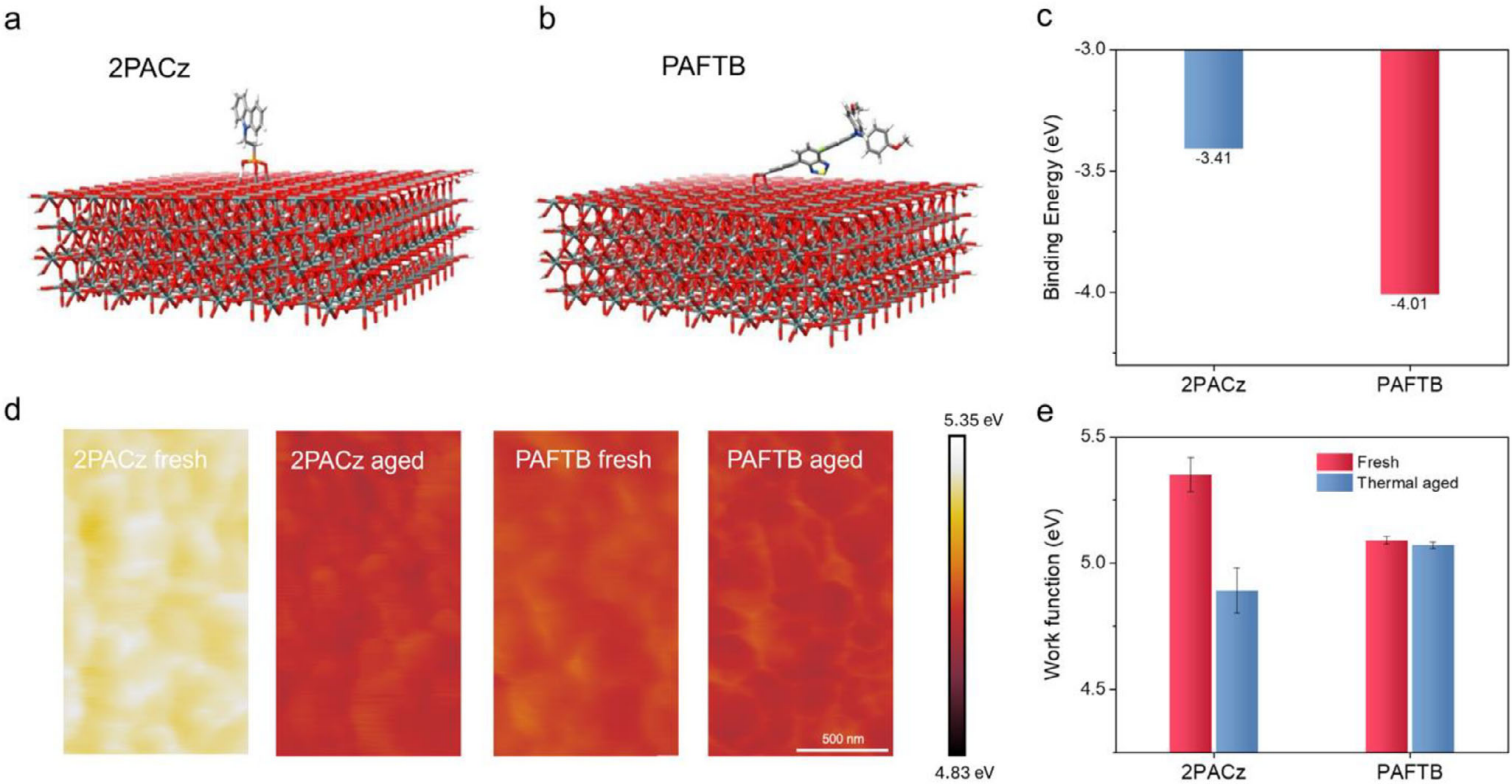
Strong binding between SAM and FTO. a) Optimized configuration of 2PACz on SnO_2_ slab. b) Optimized configuration of PAFTB on SnO_2_ slab. c) Binding energies of 2PACz and PAFTB on the SnO_2_ surface. d) KPFM‐based work function maps of fresh and thermal‐aged SAM‐modified FTO. Aging condition: 85 °C at 50% RH for 48 h. e) Work function of the fresh and thermal‐aged SAM‐modified FTO.

To study further the origins of the improved interface contact with PAFTB, we performed density functional‐based tight binding calculations to estimate the SAM adsorption on FTO (**Figure** **2**a–c). The configuration was optimized by rotating the molecules on the tin oxide (SnO_2_) slab to obtain the maximum binding energy (Figure S3, Supporting Information). The presence of the thiadiazole group in PAFTB leads to a tilted molecule that enables interaction of oxygen in the carboxyl and nitrogen in the thiadiazole to interact with SnO_2_ simultaneously. This results in a higher binding energy of 4.01 eV with PAFTB on SnO_2_, compared to 3.41 eV that was calculated with 2PACz, suggesting that PAFTB molecules can bind more strongly to the FTO substrate.

We utilized Kelvin probe force microscopy (KPFM) to investigate the desorption of SAMs by measuring the changes in work function difference between fresh and aged samples.^[^ ^13^ ^]^ After aging at 85 °C for 48 h with a relative humidity of about 50%, the work function of the 2PACz‐modified FTO decreased from 5.35 to 4.87 eV, approaching the value of bare FTO substrates (Figure S9, Supporting Information).^[^ ^38^, ^39^ ^]^ In contrast, the PAFTB‐modified FTO exhibited minimal change, with work functions of 5.09 eV for fresh samples and 5.07 eV for aged samples (Figure 2d,e). This minor change in the work function suggests that PAFTB binds more strongly to the FTO substrate and demonstrates enhanced stability under thermal aging. This is consistent with the computational results, where PAFTB shows greater binding on FTO compared to 2PACz.

The interfacial adhesion measurements were also conducted on the aged samples. A minor increase in G_c_ was observed after aging for the PAFTB‐based sample, while a substantial increase for 2PACz (Figure S4, Supporting Information). The increase in G_c_ for 2PACz samples is attributed to direct contact between the perovskite and FTO, where the FTO/perovskite interface exhibits enhanced fracture energy with a G_c_ of 7.85 J·m^−2^. This indicates that 2PACz undergoes greater desorption from the substrate. In contrast, PAFTB forms a more robust and thermally stable bond with FTO, offering superior interfacial stability under aging conditions.^[^ ^40^ ^]^

### 2.3 Enhanced Interaction with Perovskite

**Figure 3:**
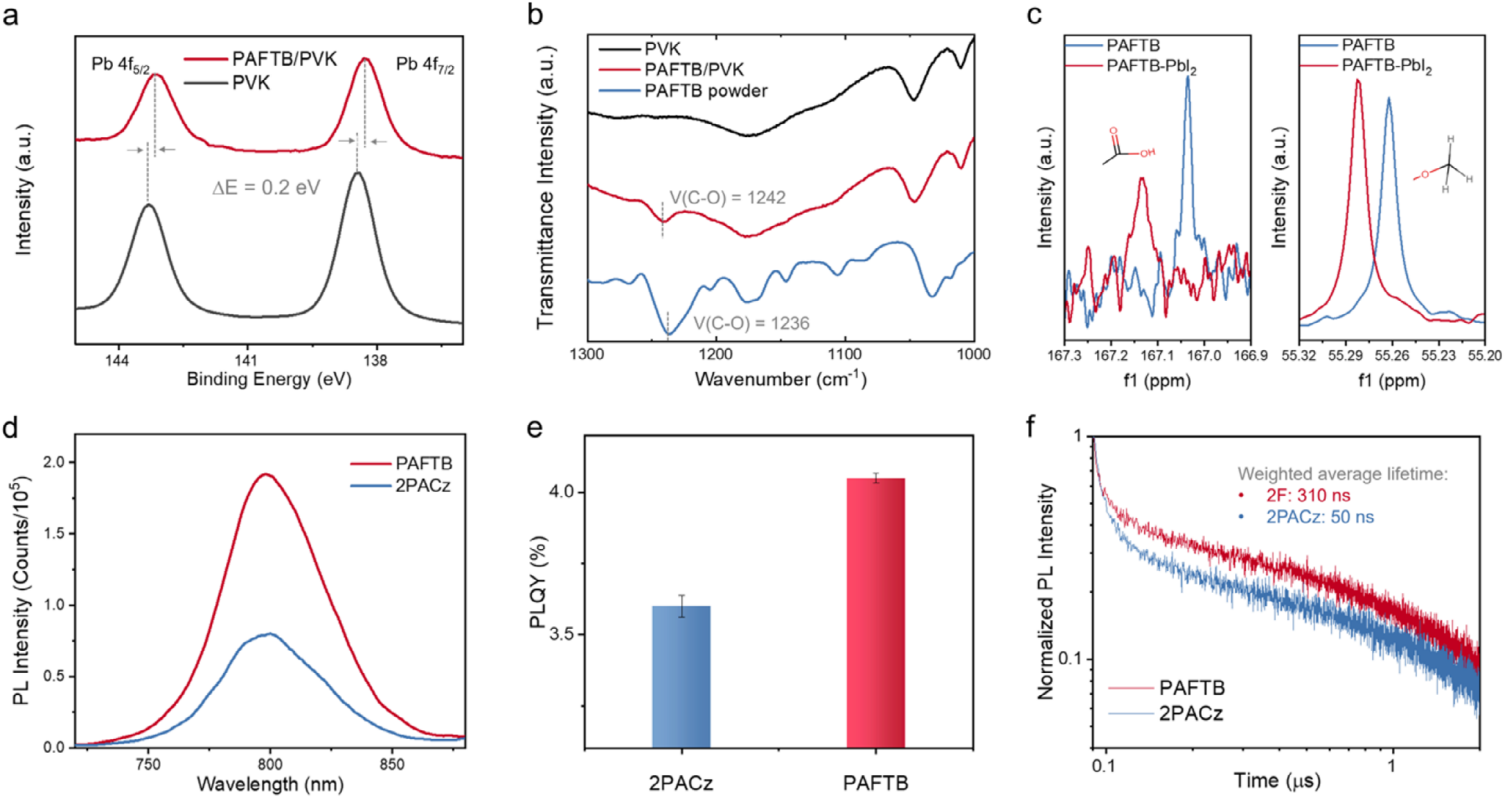
Enhanced interaction with perovskite. a) Pb 4f XPS spectra of pure perovskite film and PAFTB‐modified perovskite film. b) FTIR spectra of pure perovskite, PAFTB‐modified perovskite, and pure perovskite. c) C^13^ NMR spectra of PAFTB and V with PbI_2_, showing the interaction of the carboxyl group and methoxy group on PAFTB with Pb^2+^. d) Steady–state PL spectra of perovskite grown on PAFTB and 2PACz. e) PLQY of perovskite grown on PAFTB and 2PACz. f) TRPL of perovskite grown on PAFTB and 2PACz.

We then explored how the molecular structures influence their interaction with the perovskite layer.^[^ ^41^, ^42^, ^43^ ^]^ For the sample preparation, SAM molecules were dissolved in isopropyl alcohol (IPA) and then deposited onto the perovskite. The X‐ray photoelectron spectra of Pb 4*f* validate the interaction between SAM and perovskite (**Figure** **3**a). Compared with the control perovskite film, both characteristic Pb 4*f*_5/2_ and Pb 4*f*_7/2_ peaks of the PAFTB‐passivated perovskite film shifted to lower binding energy by 0.2 eV. This shift indicates an increased electron density at Pb^2+^, which could be caused by the coordination with the fluorine (F) and methoxy (─OCH_3_) terminal groups on PAFTB.^[^ ^31^, ^44^ ^]^ This passivation effect is absent in 2PACz, which lacks such functional groups, as indicated by the increased Pb 4*f* binding energy (Figure S5, Supporting Information).

Attenuated total reflectance Fourier‐transform infrared spectroscopy (ATR‐FTIR) was performed to study the interaction between functional groups and perovskite. C─O stretch signals from the ─OCH_3_ groups were detected in the perovskite/PAFTB samples with the C─O stretch peak (≈1240 cm^−1^) shifting to a higher wavenumber compared to neat PAFTB, which confirms the interaction between PAFTB and perovskite (Figure 3b). Additionally, solution nuclear magnetic resonance spectroscopy (NMR) was performed by dissolving PAFTB and PbI_2_ powders in deuterated dimethyl sulfoxide (DMSO) to estimate the interaction of SAM with Pb^2+^ cations (Figure 3c). The C‐13 signals from methoxy (─OCH) and carboxyl (─COOH) groups of PAFTB molecules shifted downfield after mixing with PbI_2_, indicating a decrease in electron density around these specific carbons due to changes in the chemical environment. The decrease in the electron density is attributed to the nearby oxygen atoms donating electron density to Pb^2+^ cations. These results demonstrated that the oxygen atoms in the ─OCH groups interact with Pb^2+^ cations, while the ─COOH group, expected to serve as the anchoring group binding to FTO, may also potentially interact with Pb^2+^, in cases where the HTL consists of multiple molecular layers.^[^ ^45^, ^46^, ^47^ ^]^

We further investigated the charge recombination kinetics of perovskite films based on different SAMs. The perovskite (PVK) film grown on the PAFTB SAM layer exhibits a higher PL intensity compared to the one grown on 2PACz, with both emission wavelengths centered at 800 nm (Figure 3d). Photoluminescence quantum yield (PLQY) measurements reveal that the PAFTB/PVK sample has a higher PLQY than the 2PACz/PVK sample, quantifying and confirming the presence of fewer trap states and reduced non‐radiative recombination in the PAFTB/PVK film (Figure 3e).^[^ ^32^, ^48^ ^]^

The time‐resolved photoluminescence (TRPL) spectroscopy via time‐correlated single photon counting shows that a longer carrier lifetime is observed with the PAFTB/PVK sample, indicating improved charge transport, which is attributed to the improved quality of the buried interface (Figure 3f). These enhancements are also confirmed in the scanning electron microscopy (SEM) images, which revealed that PAFTB promotes perovskite crystal growth, leading to a larger average grain size compared to 2PACz (Figure S6, Supporting Information).^[^ ^49^, ^50^ ^]^ This observation is further supported by the improved crystallinity of the perovskite films via X‐ray diffraction (XRD) measurements (Figure S7, Supporting Information).^[^ ^51^ ^]^ Contact angle measurements were performed using water. Both TAFTB and 2PACz are hydrophilic SAMs with contact angles smaller than 90° (Figure S8, Supporting Information). TAFTB shows a slightly lower contact angle than 2PACz, suggesting that functional groups on TAFTB, such as methoxy groups, increase hydrophilicity. This enhancement of wetting on TAFTB is beneficial for perovskite deposition and crystal growth.^[^ ^52^, ^53^ ^]^ These findings suggest that SAMs play a critical role not only in enhancing interface contact but also in facilitating perovskite film growth.

### 2.4 Device Performance

**Figure 4:**
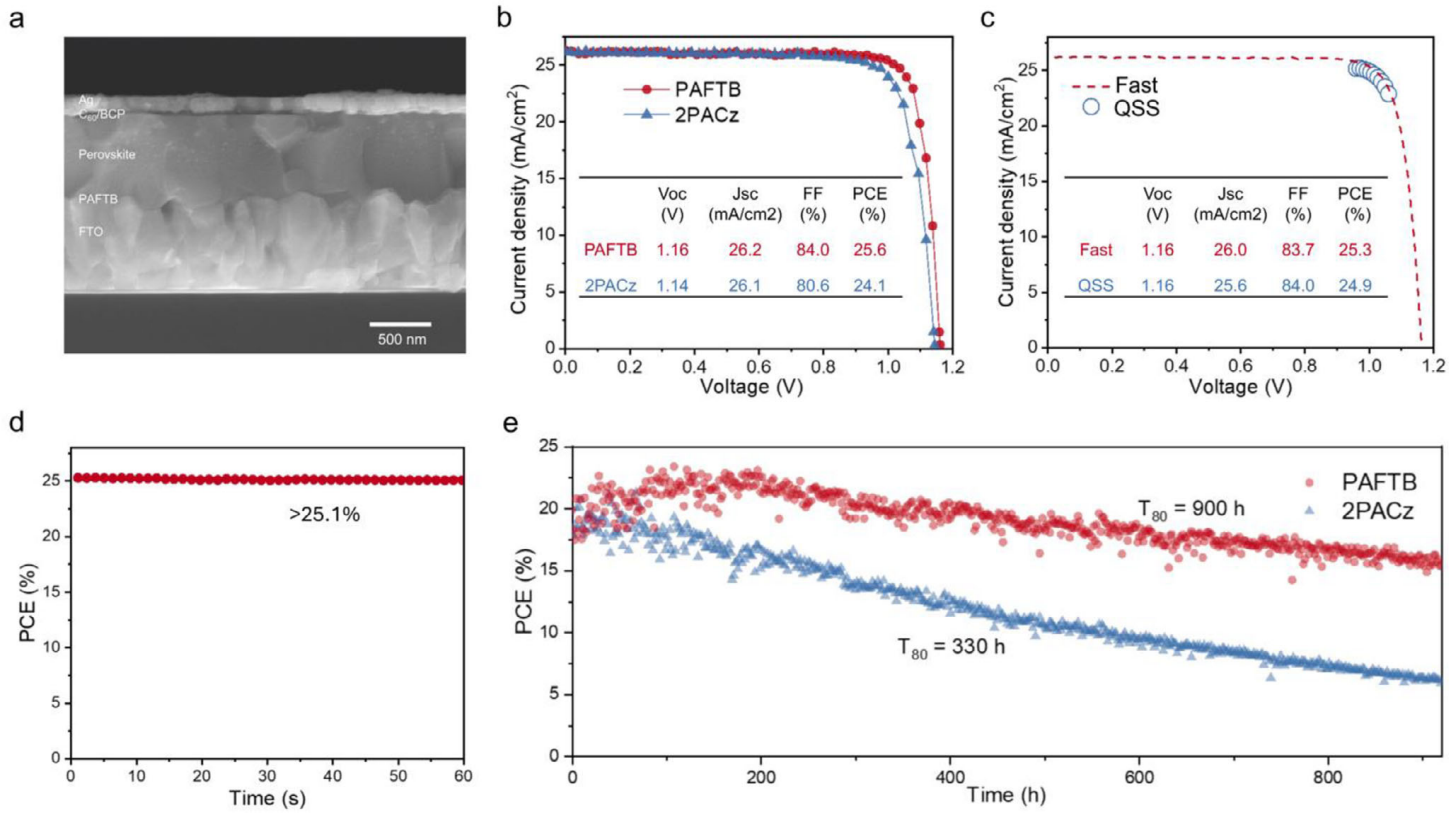
Device performance and stability. a) Cross‐section SEM image of PAFTB device. b) *J*–*V* curves of champion PAFTB and 2PACz PCSs. c)Fast scan and QSS *J–V* curve of PAFTB‐based device certified at NREL. d) Stabilized power output of the PAFTB device. e) MPP tracking of PAFTB and 2PACz PCSs under 85 °C.

We demonstrated the application potential by fabricating inverted PSCs with structures of FTO/SAMs/perovskite/passivation/C_60_/bathocuproine (BCP)/Ag (**Figure** **4**a).^[^ ^3^ ^]^ The best‐performing devices were fabricated using PAFTB, achieving a fast‐scan PCE of 25.6%, compared to 24.1% for 2PACz. The fill factor (FF) improved from 80.6% to 84.0%, and the open‐circuit voltage (*V*_oc_) increased from 1.15 to 1.16 V (Figure 4b), which we attribute to the reduced nonradiative recombination due to the improved buried interface interaction that provides effective passivation effects. Further, we attribute the good performance of the target device to favorable energetic alignment for hole transfer, as shown in Figure S9 (Supporting Information), and the dipole effect of the SAM layer. For TPFB, the dipole is oriented with its positive end toward the perovskite and negative end toward the FTO, generating a built‐in field that promotes hole transport while blocking electron injection. This reduces interfacial recombination and enhances charge extraction (Figure S10, Supporting Information). Although PAFTB exhibits some parasitic absorption (Figure S11, Supporting Information), its impact on device performance is negligible due to the ultrathin nature of the SAM layer and the additional advantages PAFTB provides. The PAFTB device was certified at the National Renewable Energy Laboratory (NREL) following the asymptotic maximum power scan protocol, with a reported fast scan PCE of 25.3% and quasi‐steady‐state (QSS) PCE of 24.9% (Figure 4c; Figure S12, Supporting Information), which is close to the record value of the state‐of‐the‐art devices certified by NREL. The external quantum efficiency (EQE) was also reported for the certified device (Figure S12, Supporting Information). Additionally, the stabilized power output of PAFTB devices, measured by maximum power point (MPP) tracking, reached 25.1% for over 60 s, indicating stable and reliable operation (Figure 4d). The minimal hysteresis between forward and reverse scans also reflects stable PSC performance (Figure S13, Supporting Information). 1 cm^2^ devices incorporating PAFTB were also fabricated, demonstrating a best PCE of 24.3% (Figure S14, Supporting Information).

We assessed the device stability by tracking the MPP of the encapsulated devices at 85 °C under 1‐sun illumination. A T_80_ test was performed, where T_80_ is defined as the time at which the device has degraded to 80% of the initial efficiency. The PAFTB device has a T_80_ lifetime of 900 h, while the 2PACz device has a shorter T_80_ lifetime of 330 h (Figure 4e). The improved operating stability of PAFTB devices indicates that PAFTB constructs a more stable buried interface under high temperatures. We attribute the overall improvement to enhanced interfacial adhesion,^[^ ^14^, ^16^ ^]^ along with the intrinsic D‐𝜋‐A structure of PAFTB, which suppresses non‐radiative recombination, facilitates hole transport, and provides a better crystallization of perovskite.

## 3 Conclusion

In this study, we demonstrated that applying a high dipole moment and D‐𝜋‐A structure molecule, PAFTB as HTL, substantially improves the interfacial adhesion in inverted PSCs. Compared to traditional carbazole‐based SAMs, PAFTB exhibited stronger binding with FTO and more effective interactions with perovskite, leading to improved buried FTO/perovskite interface quality and superior optoelectronic properties. As a result, these PSCs achieved an NREL‐certified QSS PCE of 24.9% and excellent operating stability, with a *T*_80_ of 900 h at 85 °C. This work highlights SAMs with high dipole moments as promising next‐generation charge transport materials for enhancing the thermal stability of perovskite optoelectronics.

## 4 Experimental Section

### Materials

[2‐(9H‐Carbazol‐9‐yl)ethyl]phosphonic Acid (2PACz), PbI_2_ (99.99%), and PbCl_2_ (99.99%) were purchased from TCI America. 4‐(7‐(4‐(bis(4‐methoxyphenyl)amino)‐2,5‐difluorophenyl)benzo[c][1,2,5]thiadiazol‐4‐yl)benzoic acid (PAFTB) is purchased from DYENAMO. Formamidinium iodide (FAI), methylammonium iodide (MAI), methylammonium chloride (MACl), and propane‐1,3‐diammonium iodide (PDAI2) were purchased from Greatcell Solar Materials. Cesium iodide (CsI, 99.99%), 3‐(methylthio)propylamine (3MTPA, 97%), N, N‐dimethylformamide (DMF, anhydrous, 99.8%), dimethyl sulfoxide (DMSO, anhydrous, 99.9%), isopropanol (anhydrous, 99.5%), ethanol (anhydrous, 99.5%), chlorobenzene (anhydrous, 99.8%), anisole (anhydrous, 99.7%) were purchased from Sigma–Aldrich. 3‐(methylthio)propylamine hydroiodide (3MTPAI) is obtained from reacting with hydroiodic acid with a molar ratio of 1:1.The solution was stirred in an ice bath for 2 h, then subjected to rotary evaporation at 50 °C until a white solid formed. The solid was washed several times with diethyl ether and vacuum‐dried to yield the corresponding ammonium halide salts.^[^ ^3^ ^]^ C_60_ and bathocuproine (BCP) were purchased from Xi'an Polymer Light Technology. All the materials were used as received without any purification.

### Device Fabrication

Normal bandgap perovskite precursor of 1.53 m Cs_0.05_FA_0.85_MA_0.1_PbI_3_ was dissolved in mixed DMF and DMSO solvent (4:1 v/v) with an additional 5 mol% MAPbCl_3_. Fluorine‐doped tin oxide (FTO) substrates were prepared by sonicating with detergent, deionized water, acetone, and isopropanol for 15 min sequentially. The substrates were then dried with nitrogen gas and underwent ultraviolet ozone treatment for 30 min. The hole transport layer (HTL) was deposited onto the cleaned FTO substrate by spin coating 100 µ*L* of PAFTB (1.5 mg mL^−1^) or 2PACz (0.5 mg mL^−1^) dissolved in ethanol at 4500 r.m.p. for 25 s, ​followed by annealing at 100 °C for 10 min. After cooling down, 60 µ*L* of perovskite precursor was deposited onto the HTL layer and spin‐coated at 1000 r.m.p. for 5 s (acceleration rate: 200 r.p.m./s) and then 5000 r.m.p. for 25 s (acceleration rate: 2000 r.p.m./s), with 150 µ*L* of anisole dropped at the 10 s of the second step. The films were annealed at 100 °C for 15 min. When the films cooled down, the post‐treatments followed the reported bi‐molecular passivation method;^[^ ^3^ ^]^ dissolving 10 mm 3MTPAI and 3 mm PDAI_2_ in isopropanol/chlorobenzene (1:1 v/v), the resulting mixture was dynamically spin‐coated onto the perovskite film at 4500 r.m.p. for 25 s, followed by annealing at 100 °C for 5 min. The electron transport layer (HTL) consisted of thermally evaporated 30 nm C_60_ on the perovskite films, followed by evaporating 7 nm BCP as a hole‐blocking layer under a vacuum of ≈10^−7^ torr. Subsequently, a 140 nm Ag electrode was evaporated under a vacuum of ≈10^−7^ Torr.

### Characterizations_Current–Density–Voltage (*J–V*) Test

The *J–V* characteristics were measured under nitrogen gas in a glovebox at room temperature with a Keithley 2400 source meter. A Sciencetech A1 Light Line Class AAA solar simulator was used to simulate AM 1.5G irradiation with an Xe arc lamp. The light intensity was calibrated with a Sciencetech SCI‐REF‐Q silicon cell. The scanning step was 20 mV and the scanning rate was 70 mV s^−1^. An opaque metal mask was used to define the active area, which was 0.049 cm^2^.

### Characterizations_Operational Stability Test

The operational stability tests were performed using Flexium under nitrogen gas with all devices encapsulated. Encapsulation was carried out using a glass cover slide, sealed with ultraviolet‐curable adhesive (Lumtec LT‐U001). The light intensity is 1‐sun illumination. The testing temperature was 85 °C and the relative humidity was ≈50%.

### Characterizations_Interfacial Adhesion (Fracture Energy) Measurements

The double cantilever beam (DCB) samples adopted a structure of glass/FTO/SAM/perovskite/PMMA/Ag/epoxy/glass. The epoxy used was Loctite epoxy instant mix 5 min, which was applied to the top substrate (bare glass) and bonded to the bottom glass substrate. Specific details of the testing method can be found elsewhere.^[^ ^54^, ^55^ ^]^ Briefly, a pre‐crack was introduced into the DCB sample along the orientation of the crack propagation by inserting the tip of a razor blade into the two glass substrates. The resulting pre‐crack can protect the DCB sample from excessive tensile loads to initiate crack propagation. By using the delaminator system (DTS, USA), the DCB samples were mounted and loaded in tension at a displacement rate of 1 µm ^−1^s. When a unit of well‐defined opening (mode I) fracture occurred, the DCB sample was unloaded and loaded again to gradually propagate the crack in the sample until a complete separation was achieved for the two glass substrates, suggesting the completion of crack propagation. In the measurement, the load (*P*) – displacement (Δ) curves were continuously recorded and used to extract the fracture energy (*G_c_*), which can be calculated by the following equation:

(1) $$\begin{array}{c} \;G_{c}=\frac{12P_{c}^{2}a^{2}}{B^{2}E^{\prime }h^{3}}\;{\left(1+0.64\frac{h}{a}\right)}^{2} \end{array}$$

where *P_c_* ​ is the critical load at which deviation from linearity occurs in the *P*‐Δ curve during the loading cycle, *a* is the crack length, *B* and *h* are the width and half‐height of the sample, respectively, and *E*′ is the plane‐strain elastic modulus of the substrate. The crack length was estimated by the following compliance method:

(2) $$\begin{array}{c} a={\left(\frac{d\Delta }{\mathrm{dP}}\cdot \frac{{\mathrm{BE}}^{\prime }h^{3}}{8}\right)}^{\frac{1}{3}}-0.64h \end{array}$$

where **Δ** is the displacement and ***P*** is the applied load. The ***G***_***c***_ tests were performed under a laboratory air environment.

### Other Characterizations

X‐ray diffraction (XRD) was performed using a Rigaku Miniflex diffractometer with Cu Kα1 radiation. The SEM images of the perovskite film and cross‐section view were taken by JEOL JSM‐7900FLV. The accelerating voltages are (15 kV). Kelvin probe force microscopy (KPFM) images were generated using an Asylum Cypher S atomic force microscope (Oxford Instruments) with a Ti‐Ir‐coated ASYELEC.01‐R2 cantilever and k = 4 ± 0.5 N m^−1^ (AsylumResearch). Scans were performed over 2 × 2 µm at 512 pixels and 1 Hz in a two‐pass nap method, the first pass in tapping mode and the second in KPFM mode with a tip potential of 3 V and surface clearance of 5 nm. KPFM measurements were performed after calibrating the tip using a reference surface with a known work function—100 nm thick electron beams evaporated Au, 5.3 eV. Nuclear magnetic resonance (NMR) spectroscopy was measured using Bruker Avance III™ HD 500 MHz. The photoluminescence quantum yield (PLQY) was measured using a LuQY Pro System (Quantum Yield Berlin) under excitation from a 532 nm, 100 mW laser. Photoluminescence spectroscopy (PL) and Time‐resolved photoluminescence (TRPL) measurements were performed using an Edinburgh FS5 spectrofluorometer with 450 and 373 nm excitation, respectively. Attenuated total reflectance‐Fourier transform infrared (ATR‐FTIR) spectra were measured with a Nexus 870 spectrometer. X‐ray photoelectron spectroscopy (XPS) spectra were taken using a Nexsa G2 X‐Ray Photoelectron Spectrometer. Photoemission Yield Spectroscopy in Air (PYSA) measurements were taken on a Riken–Keiki. Contact angle measurements were carried out using a VCA Optima XE. UV–vis measurements were conducted on a Shimadzu 3600 UV–vis spectrometer.

### Computational Details

The structure of the tetragonal SnO_2_ unit cell (space group: P4_2_/mnm) was optimized by density functional theory (DFT) calculation in VASP with GGA and optB86b‐vdW.^[^ ^56^, ^57^, ^58^, ^59^, ^60^ ^]^ A 4‐layered supercell was constructed to study the binding of 2PACz and PAFTB (separately optimized at a B3LYP / Def2‐SVP level in Q‐Chem) on the SnO2 (110) surface.^[^ ^61^ ^]^ Density functional tight‐binding calculations were performed with the GFN1‐xTB Hamiltonian, D3 dispersion correction, and BJ damping in DFTB+ to study the binding energy of the molecule on the surface.^[^ ^62^, ^63^, ^64^ ^]^

(3) $$E_{BE}=E_{SnO_{2}+mol}\;-\left(E_{SnO_{2}}+E_{mol}\right)$$

where $E_{SnO_{2}+mol}$, $E_{SnO_{2}}$, and *E_mol_* are the energies of the system for the SnO_2_ slab with the molecule attached, the isolated SnO_2_ slab, and the isolated molecule, respectively.

Multiple configurations were generated with the molecule being bent different amounts with respect to the surface, and the Boltzmann‐averaged binding energy (*E*_*BE*(*B*)_) was calculated as

(4) $$E_{BE\left(B\right)}=\frac{\sum_{i}E_{BE\left(i\right)}e^{\frac{-E_{\left(i\right)}}{k_{B}T}}}{\sum_{i}e^{\frac{-E_{\left(i\right)}}{k_{B}T}}}$$

where *E*_*BE*(*i*)_ is the binding energy calculated at the i^th^ configuration, *k_B_* is the Boltzmann constant, and *T* is the temperature (298 K).

## Conflict of Interest

The authors declare no conflict of interest.

## Supporting information



Supporting Information

## Data Availability

The data that support the findings of this study are available from the corresponding author upon reasonable request.
