# Supplementary material for: Electrostatically Enhanced Buried Interface Binding of Self‐Assembled Monolayers for Efficient And Stable Inverted Perovskite Solar Cells
Source: Adv Mater. 2025 Aug 11;37(43):e08740. doi: 10.1002/adma.202508740 (PMC12574631; doi:10.1002/adma.202508740)
Supplement: Supplementary file 1 — Supporting Information [file ADMA-37-e08740-s001.docx]

**Supporting information**

# **Electrostatically enhanced buried interface binding of self-assembled monolayers for efficient and stable inverted perovskite solar cells**

Chuying Huang^1^†, Yi Yang^1^†, Cheng Liu^1^, Hao Chen^1^, Subhajyoti Chaudhuri^1^, Woo Cheol Jeon^1^, Muzhi Li^4^, Nicholas Rolston^4^, Abdulaziz S. R. Bati^1^, Isaiah W. Gilley^1^, Boran Kumral^5^, Peter Serles^5^, Tobin Filleter^5^, George C. Schatz^1^, Mercouri G. Kanatzidis^1^, Bin Chen^1^*, Lin X. Chen^1^*, Edward H. Sargent^1,2,3^*

^1^Department of Chemistry, Northwestern University, 2145 Sheridan Rd, Evanston, Illinois 60208, USA.

^2^Department of Electrical and Computer Engineering, University of Toronto, 35 St George Street, Toronto, Ontario, M5S 1A4, Canada.

^3^Department of Electrical and Computer Engineering, Northwestern University, 2145 Sheridan Rd, Evanston, Illinois 60208, USA.

^4^Ira A. Fulton Schools of Engineering, Arizona State University, Tempe, AZ 85281, USA.

^5^Department of Mechanical and Industrial Engineering, University of Toronto, Toronto, ON M5S 3G8, Canada.

†These authors contributed equally to this work.

*Corresponding Authors: bin.chen@northwestern.edu (B.C.), l-chen@northwestern.edu (L.X.C.), ted.sargent@northwestern.edu (E.H.S.)


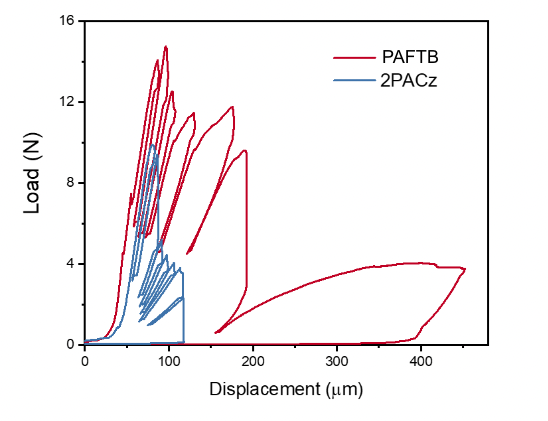


Figure S1. Load-displacement curve of the interfacial adhesion test using the DCB method.


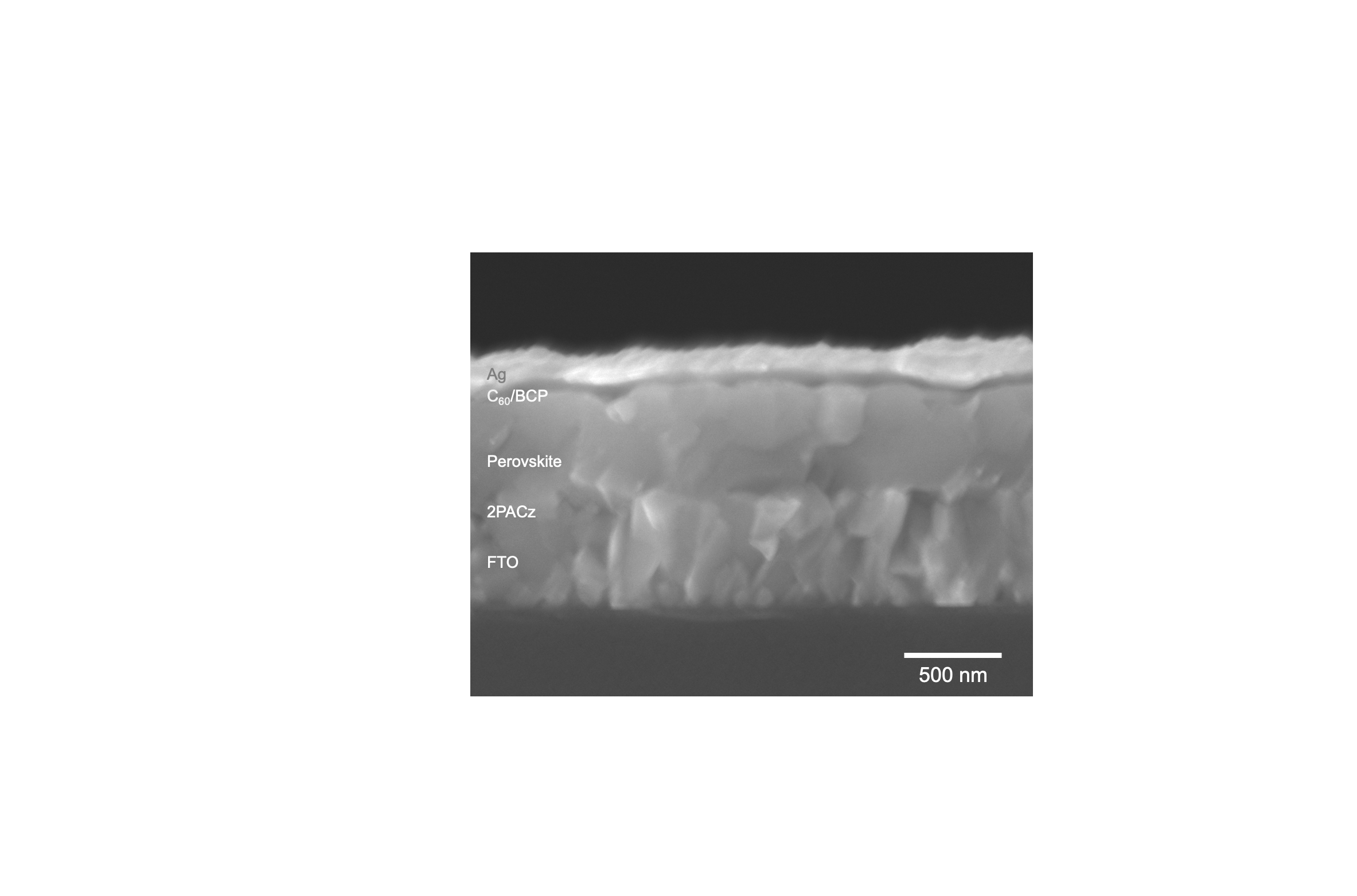


Figure S2. Cross-section SEM image of 2PACz device.

Figure S3. The 2PACz and PAFTB molecules were rotated with respect to the SnO_2_ slab in 5^o^ increments and binding energies for each configuration were calculated.


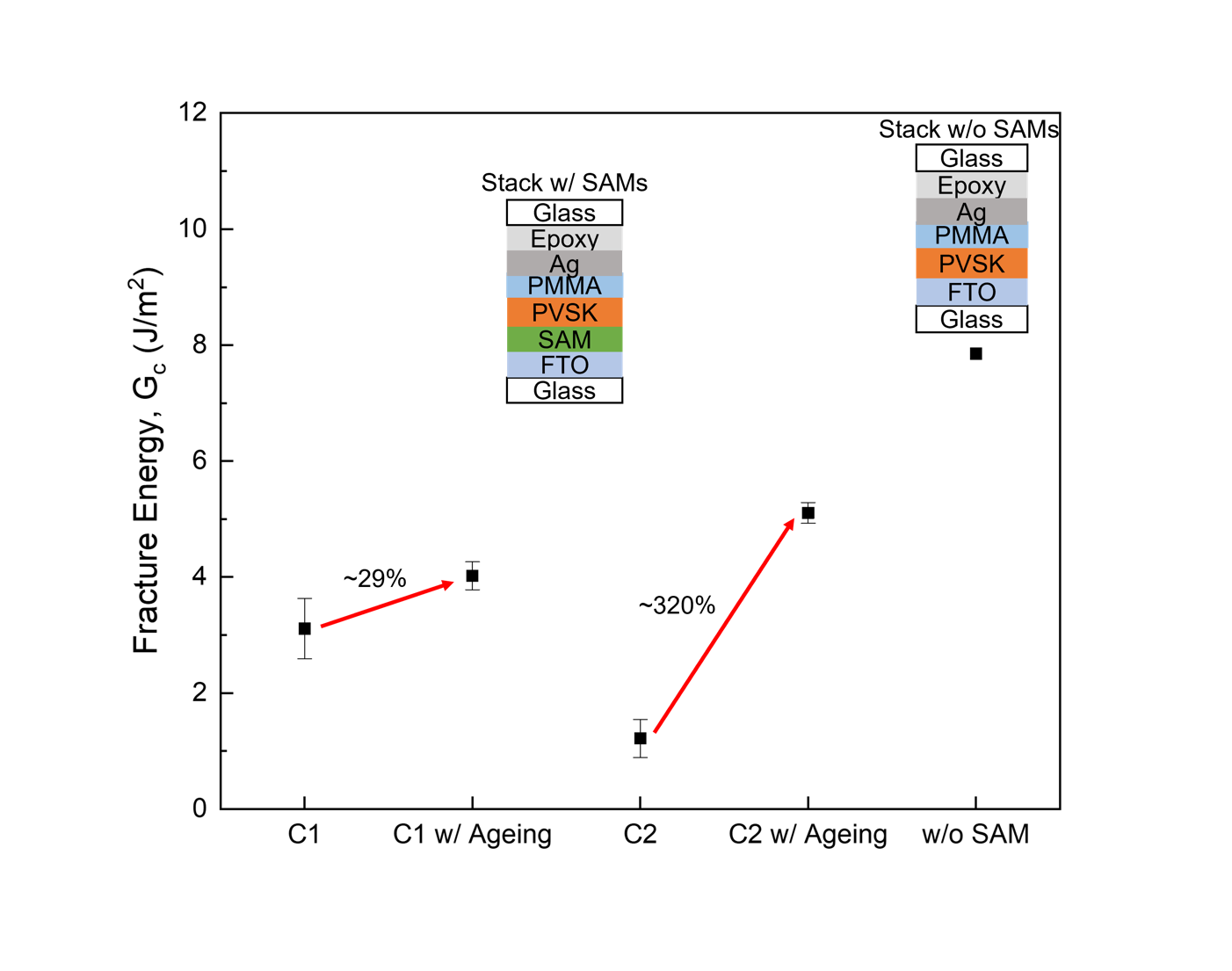


Figure S4. G_c_ results of samples with SAMs before and after aging, and without SAMs. C1 represents PAFTB and C2 represents 2PACz. The insets illustrate the layer stacks.


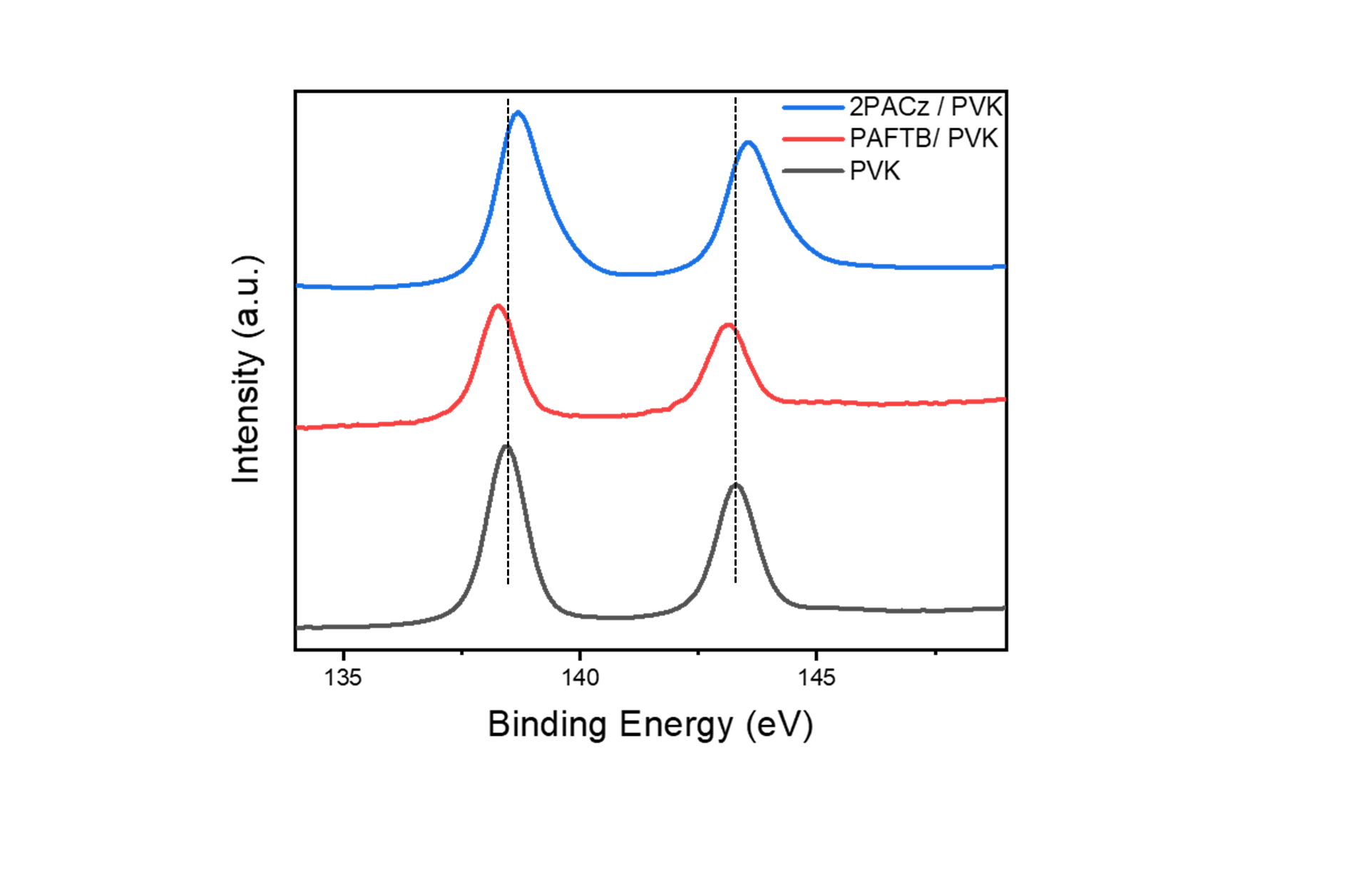


Figure S5. XPS spectra of Pb 4f core levels of pure perovskite and SAM-modified perovskite.


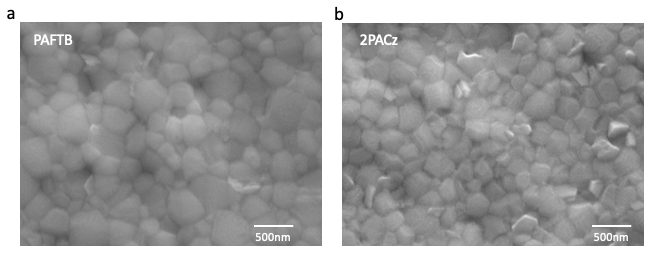


Figure S6. SEM images of perovskite films grown on (a)PAFTB and (b)2PACz.


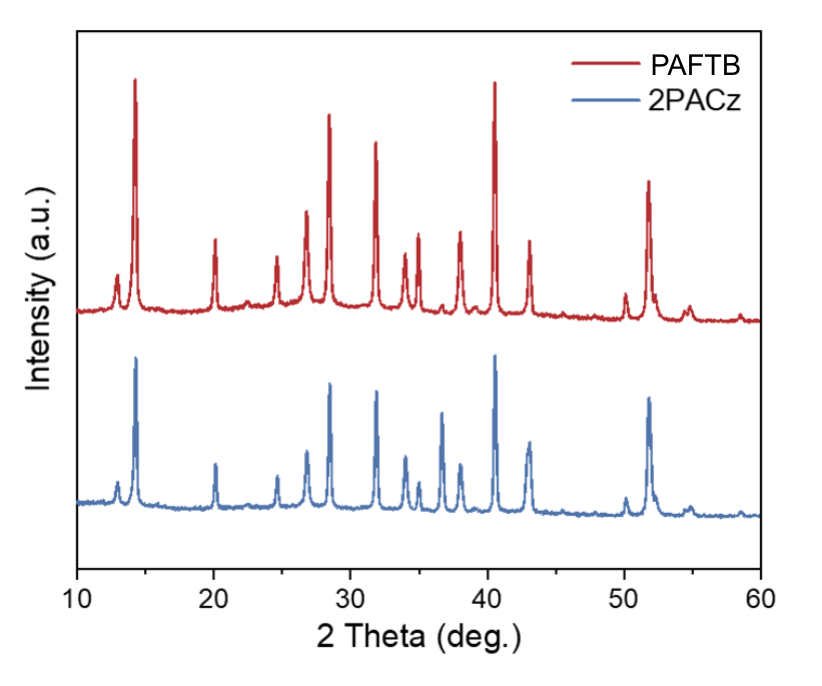


Figure S7. XRD of perovskite films grown on PAFTB and 2PACz.


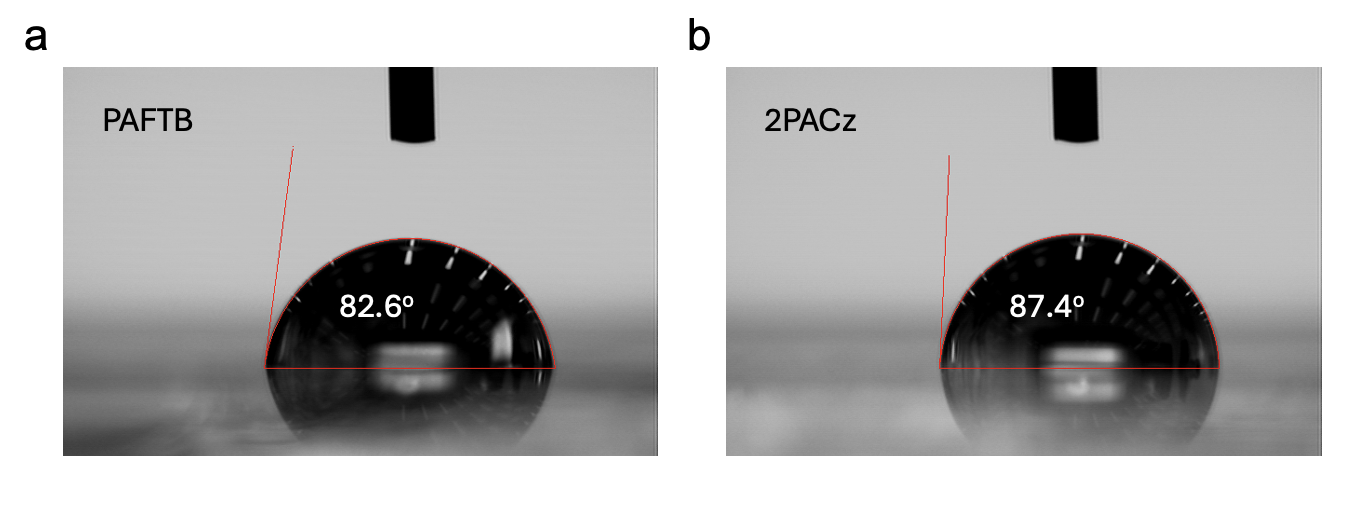


Figure S8. Contact angle measurements a) PAFTB and b)2PACz modified FTO.


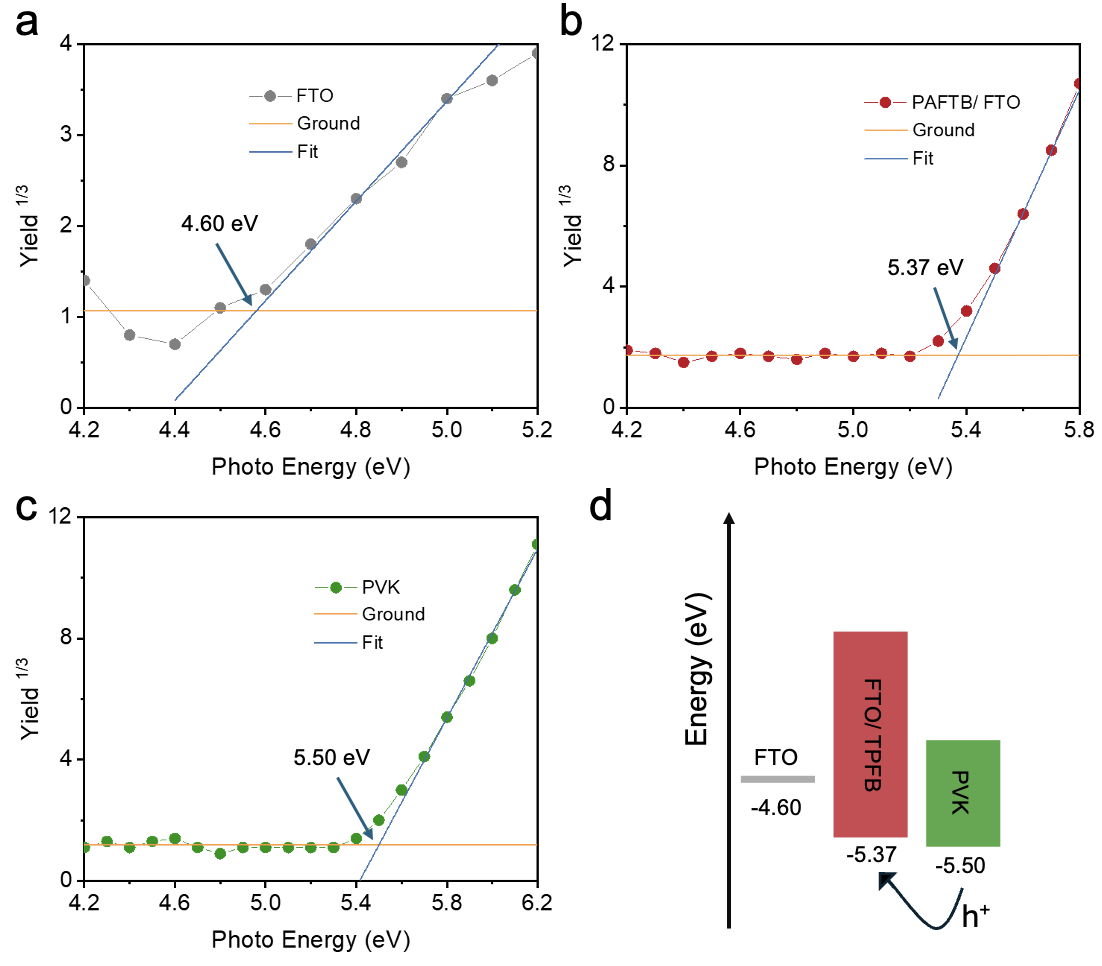


Figure S9. PYSA measurements a) FTO, b) PAFTB deposited on FTO, and c) perovskite. d) Energetic alignments of perovskite, HTL, and FTO.


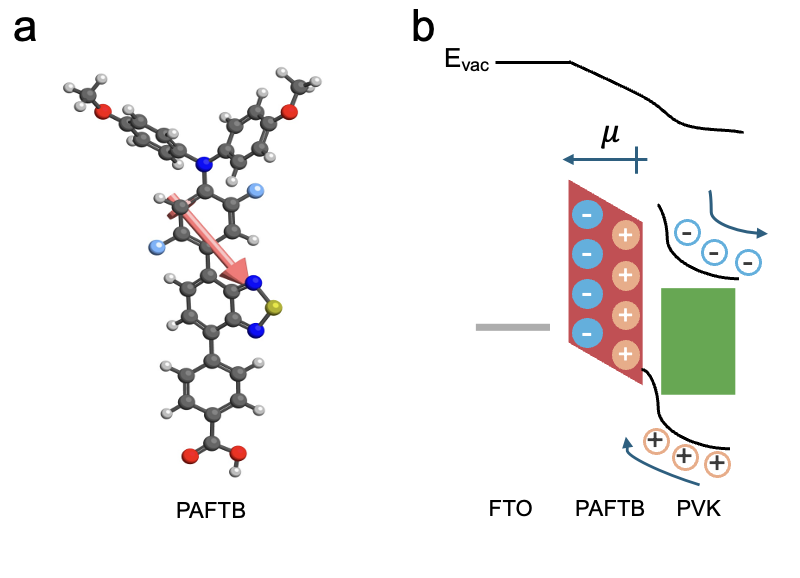


Figure S10. a) Dipole moment vector in PAFTB molecule. b) Dipole interlayer of SAMs can create a built-in electric field, which facilitates hole transport.


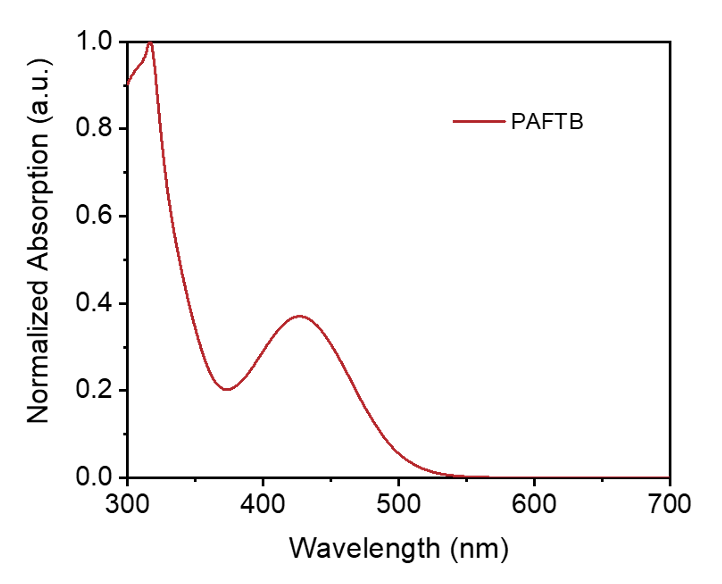


Figure S11. Absorption spectrum of PAFTB.


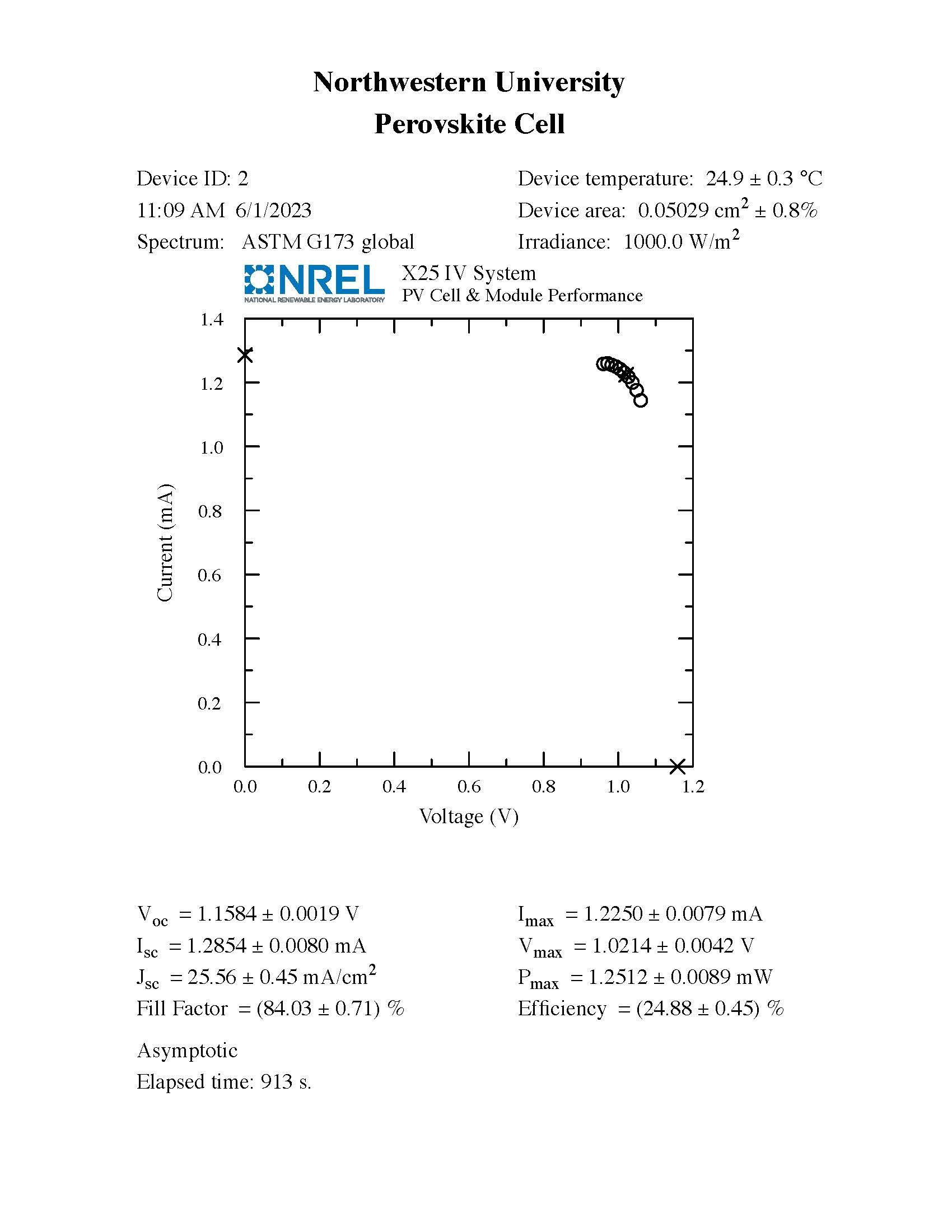

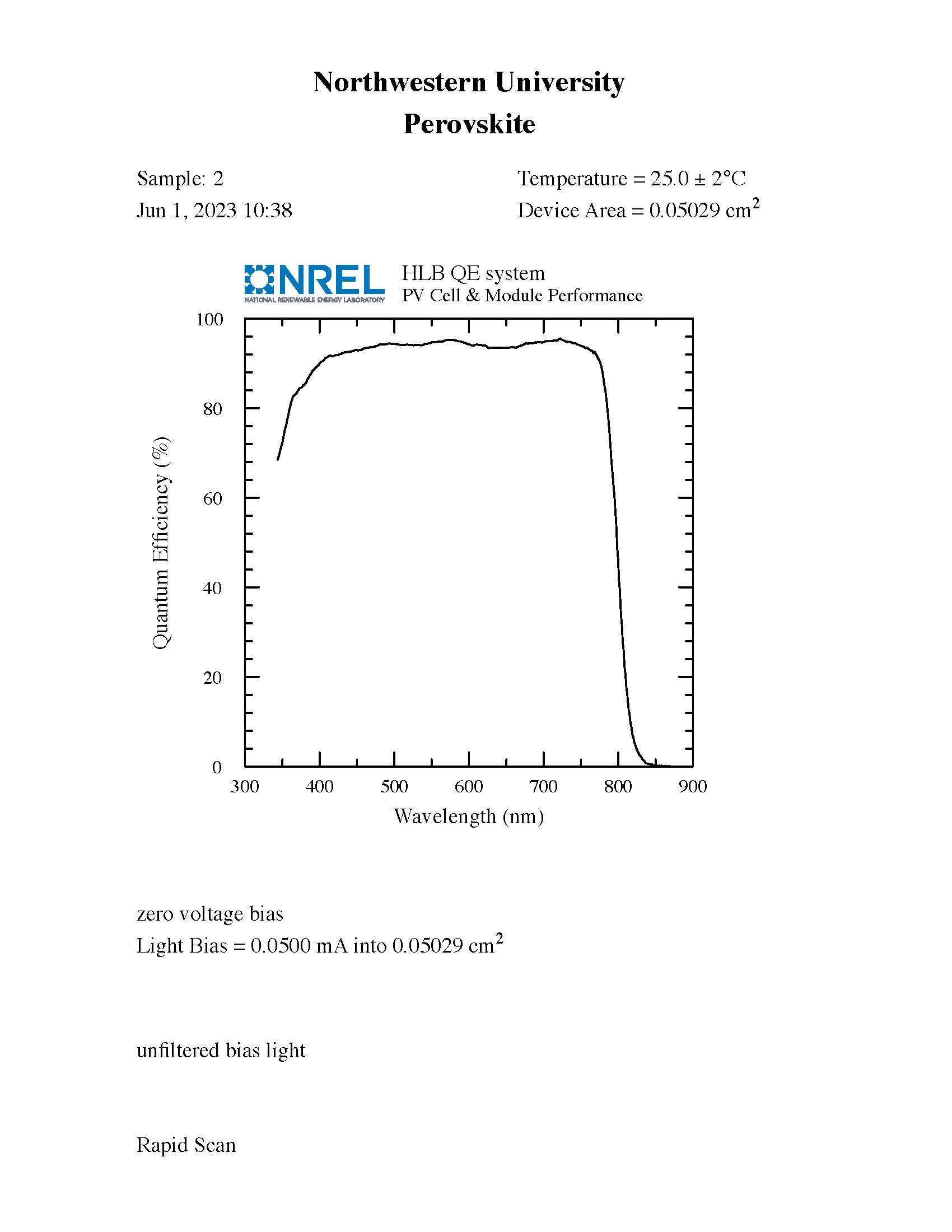


Figure S12. Certification of stabilized PV performance and external quantum efficiency (EQE) of PAFTB device measured by NREL PV Performance Group.

*
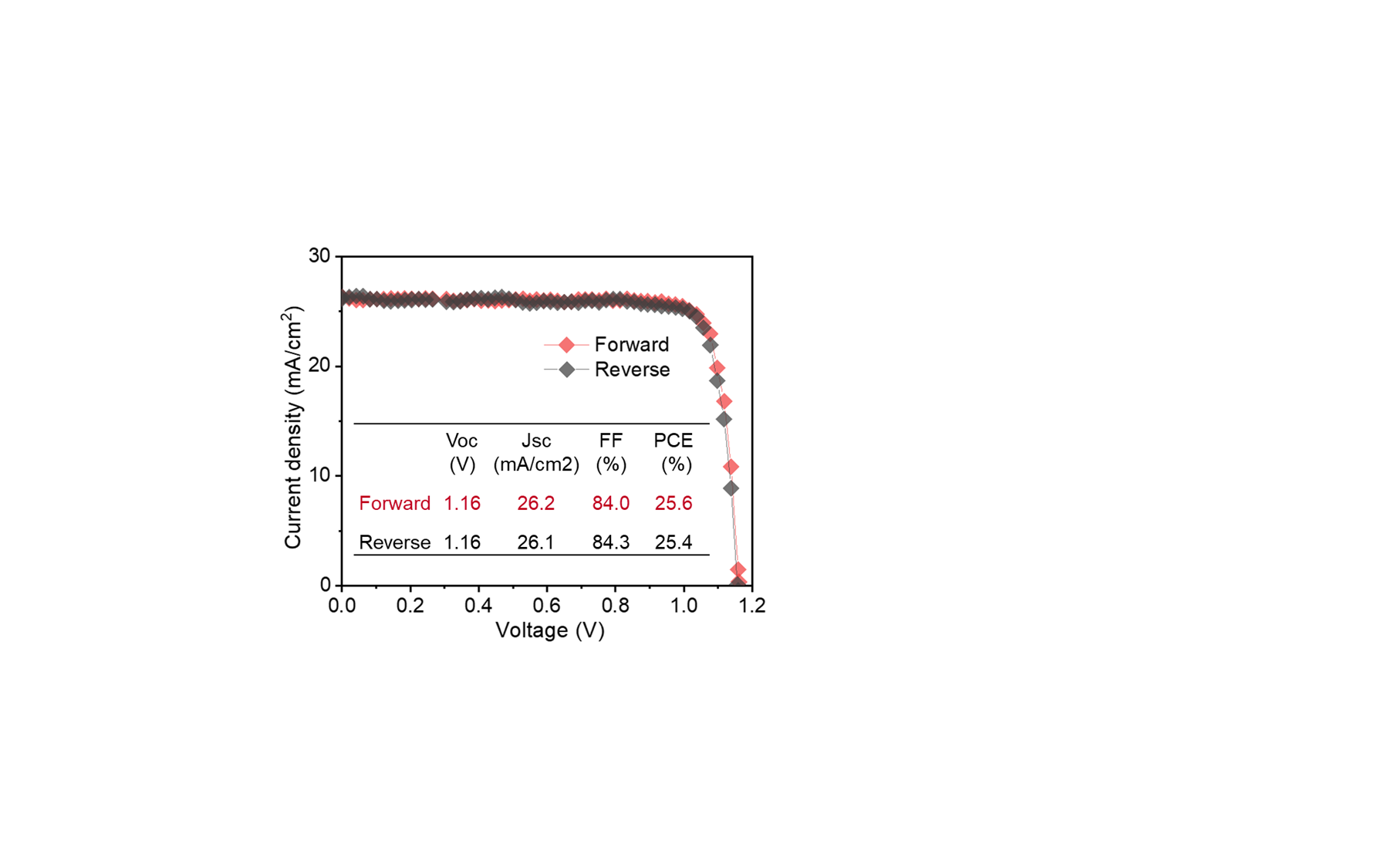
*

Figure S13. Forward and reverse scan of the champion PAFTB device (0.049cm^2^).


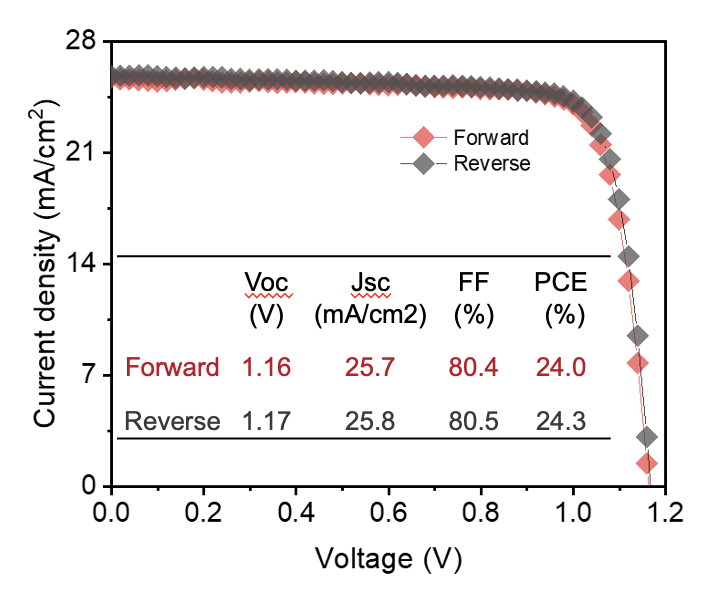


Figure S14. Forward and reverse scan of the champion large area PAFTB device (1.0 cm^2^).
